# Altered anterior cingulate glutamatergic metabolism in depressed adolescents with current suicidal ideation

**DOI:** 10.1038/s41398-020-0792-z

**Published:** 2020-04-23

**Authors:** Charles P. Lewis, John D. Port, Caren J. Blacker, A. Irem Sonmez, Bhedita J. Seewoo, Jarrod M. Leffler, Mark A. Frye, Paul E. Croarkin

**Affiliations:** 1 Department of Psychiatry and Behavioral Sciences, University of Minnesota Medical School, Minneapolis, MN USA; 2 Department of Psychiatry and Psychology, Mayo Clinic, Rochester, MN USA; 3 Department of Radiology, Mayo Clinic, Rochester, MN USA; 4 Experimental and Regenerative Neurosciences, School of Biological Sciences, University of Western Australia, Perth, WA Australia; 5 Brain Plasticity Group, Perron Institute for Neurological and Translational Research, Perth, WA Australia; 6 Centre for Microscopy, Characterisation and Analysis, Research Infrastructure Centres, University of Western Australia, Perth, WA Australia

**Keywords:** Predictive markers, Depression, Human behaviour, Molecular neuroscience, Diagnostic markers

## Abstract

The anterior cingulate cortex (ACC) is involved in emotion regulation and salience processing. Prior research has implicated ACC dysfunction in suicidal ideation (SI) and suicidal behavior. This study aimed to quantify ACC glutamatergic concentrations and to examine relationships with SI in a sample of healthy and depressed adolescents. Forty adolescents underwent clinical evaluation and proton magnetic resonance spectroscopy (^1^H-MRS) at 3 T, utilizing a 2-dimensional *J*-averaged PRESS sequence sampling a medial pregenual ACC voxel. Cerebrospinal fluid-corrected ACC metabolite concentrations were compared between healthy control (HC, *n* = 16), depressed without SI (Dep/SI−, *n* = 13), and depressed with SI (Dep/SI+, *n* = 11) youth using general linear models covarying for age, sex, and psychotropic medication use. Relationships between ACC metabolites and continuous measures of SI were examined using multiple linear regressions. ROC analysis was used to determine the ability of glutamate+glutamine (Glx) and the *N*-acetylaspartate (NAA)/Glx ratio to discriminate Dep/SI− and Dep/SI+ adolescents. Dep/SI+ adolescents had higher Glx than Dep/SI− participants (*p*_adj_ = 0.012) and had lower NAA/Glx than both Dep/SI− (*p*_adj_ = 0.002) and HC adolescents (*p*_adj_ = 0.039). There were significant relationships between SI intensity and Glx (*p*_FDR_ = 0.026), SI severity and NAA/Glx (*p*_FDR_ = 0.012), and SI intensity and NAA/Glx (*p*_FDR_ = 0.004). ACC Glx and NAA/Glx discriminated Dep/SI− from Dep/SI+ participants. Uncoupled NAA−glutamatergic metabolism in the ACC may play a role in suicidal ideation and behavior. Longitudinal studies are needed to establish whether aberrant glutamatergic metabolism corresponds to acute or chronic suicide risk. Glutamatergic biomarkers may be promising targets for novel risk assessment and interventional strategies for suicidal ideation and behavior.

## Introduction

Suicide and suicide attempts have increased among adolescents and young adults during the past two decades^1–3^. Suicide is now the second leading cause of death in young people^1,4^. Suicidal behavior accounts for a substantial and increasing proportion of pediatric hospital visits^5^. The broad spectrum of suicidal thoughts and behaviors is remarkably common in youth; large epidemiological surveys of adolescents^6,7^ estimate high prevalence of suicidal ideation (SI; 12.1–17.7%), planning (4.0–14.6%), and attempts (4.1–8.6%). Adolescence represents a critical time in the development of suicidal behavior. More than half of index suicide attempts occur by the age of 25^8^. Furthermore, childhood and adolescent suicidality predict suicidal behavior and attempts later in life^9^. Clinical practice is uninformed by neurobiological data and relies almost entirely on parent and adolescent reporting, which demonstrate a concerning lack of concordance^10^. Despite the need for objective neurobiological markers to augment the clinical assessment of suicide risk, substantial prior research in this field has not yielded reliable predictive tools^11,12^. There is a compelling need for better brain-based markers of suicide risk and interventions that target underlying brain dysfunction.

Numerous neurotransmitter and neuroendocrine systems have been implicated in suicide and suicidal behavior^13,14^. Increasing evidence indicates dysregulated glutamatergic neurotransmission in suicidal individuals. Gene association studies have linked suicidal behavior with single nucleotide polymorphisms in genes encoding subunits of the *N*-methyl-d-aspartate (NMDA) glutamatergic receptor^15,16^ and associated enzymes and transporters^16^. Postmortem studies in suicide victims have demonstrated altered expression of genes encoding NDMA and α-amino-3-hydroxy-5-methyl-4-isoxazolepropionic acid (AMPA) ionotropic receptors^17,18^, metabotropic glutamate receptors^18^, and related proteins^17,18^. Other investigations in suicide attempt survivors have linked cerebrospinal fluid (CSF) levels of endogenous NMDA receptor agonists to suicidal behavior, which change over time following suicide attempts^19^. Further evidence for the role of glutamate in suicidality arises from the effects of ketamine, an NMDA receptor antagonist, in rapidly reducing suicidal ideation^20,21^.

Concentrations of glutamate (Glu) and related metabolites, including glutamine (Gln), glutamate+glutamine (Glx), *N*-acetylaspartate (NAA), and *N*-acetylaspartylglutamate (NAAG), can be quantified noninvasively using proton magnetic resonance spectroscopy (^1^H-MRS). Prior ^1^H-MRS studies of adults with unipolar depression have found reductions in Glu^22–25^, Glx^22,26–29^, Gln^29^, and NAA^24,25^ in various cortical regions. A small number of ^1^H-MRS studies in adults have examined cortical glutamate and NAA in depressed individuals with prior suicide attempts^30–32^. However, findings have been inconsistent, possibly due to divergent populations (e.g., unipolar^32^ vs. bipolar^31^ depression), different brain regions sampled, the lack of nonsuicidal depressed control groups^30^, and variable temporal distance between suicidal behavior and ^1^H-MRS measurements. Several previous ^1^H-MRS investigations have found that adolescents with major depression have decrements in Glu^33^ and Glx^34,35^ in the anterior cingulate cortex (ACC) and reduced NAA in the ACC and medial prefrontal cortex^36^ compared to healthy youth, while more recent work has examined the neurochemical basis for symptomatologic dimensions and depressive subtypes in adolescents^37,38^. However, to our knowledge no previous spectroscopic studies have examined glutamatergic correlates of current suicidal thoughts and behaviors among depressed adolescents.

This study sought to examine ^1^H-MRS-measured cortical glutamatergic metabolism in a sample of healthy and depressed youth with and without current SI. The ACC was selected as the region of interest considering (1) the robust evidence for its role in the pathophysiology of depression in adults and youth, and (2) its role in modulation of prefrontal and limbic processes^39^, notably the evaluation of negatively valent stimuli and their salience to the self^40–44^, which is highly pertinent to suicidal thoughts and behaviors^45^. We anticipated that depressed adolescents with current SI would demonstrate elevated Glx and reduced NAA in the ACC compared to nonsuicidal depressed and healthy youth. It was also hypothesized that Glx and NAA concentrations would correlate with continuous measures of SI intensity and severity. Finally, exploratory analyses were performed to examine the ability of ACC Glx and NAA/Glx to discriminate depressed adolescents with current SI from those without SI.

## Materials and methods

### Participants

Participants were adolescents between the ages of 13 and 21 years. Depressed participants were recruited from an adolescent psychopharmacology clinic and consisted of treatment-seeking youth with depressive symptoms; those who enrolled completed study assessments and ^1^H-MRS prior to undergoing appropriate clinical care (initiation of an antidepressant medication or change to another antidepressant). Healthy control participants were recruited from pediatric primary care clinics and through community advertising. Parents or guardians of minor participants (age < 18 years) provided written informed consent, and minor participants provided written informed assent; participants 18 years or older provided written informed consent. All study procedures were approved by the Mayo Clinic institutional review board (Rochester, MN, USA).

### Clinical assessment and measures

All participants and parents/guardians underwent clinical assessment by a board-certified child and adolescent psychiatrist (P.E.C.) prior to ^1^H-MRS. This included evaluation on a semi-structured diagnostic interview, the K-SADS-PL^46^. Depressive symptom severity was rated on the Children’s Depression Rating Scale, Revised (CDRS-R)^47^ based on clinical interview of the adolescent participant and parent/guardian. Seventeen individual symptom items were rated by the clinician and summed for a total CDRS-R raw score ranging from 17 to 113. Additionally, since suicidality is assessed on the CDRS-R, an adjusted CDRS-R score was calculated by subtracting two items pertaining to suicidal and morbid ideation (items 12 and 13) from the total score to yield a measure of depressive symptom severity distinct from suicidal symptoms.

Current SI was assessed by clinical interview of participants using the Columbia Suicide Severity Rating Scale (C-SSRS)^48^. The “Severity of Ideation” and “Intensity of Ideation” subscales were used to characterize participants’ suicidal thoughts. The C-SSRS “Severity of Ideation” subscale is an ordinal scale derived from five items that assess the quality of suicidal thoughts. Severity of SI is rated as: 0 = no SI; 1 = wish to be dead; 2 = non-specific active suicidal thoughts; 3 = active SI with any method (not plan) without intent to act; 4 = active SI with some intent to act, without specific plan; 5 = active SI with specific plan and intent. The C-SSRS “Intensity of Ideation” subscale is derived from five individual items that assess intensity of suicidal thoughts across several dimensions: frequency of ideation, duration of ideation, controllability of thoughts, deterrents to suicide, and reasons for suicidal ideation. Each dimension is given a score of 0 to 5, for a total Intensity of Ideation subscale score of 0 (no SI present) to 25 (maximum intensity). Additionally, based on C-SSRS items corresponding to prior lifetime suicidal behavior (SB), participants were rated on an ordinal scale of maximal lifetime SB: 0 = no prior SB; 1 = nonsuicidal self-injurious behavior; 2 = planning or preparation for an attempt; 3 = aborted or interrupted attempt; 4 = suicide attempt.

Presence or absence of psychotropic medication use at the time of ^1^H-MRS was coded as a dichotomous variable (0 = no psychotropic medication; 1 = current psychotropic medication use).

### Group eligibility and classification

Adolescents in the healthy control group had no current or historical psychiatric diagnosis, no current or previous psychopharmacologic or psychotherapeutic treatment, and had depression severity raw scores less than 30 on the CDRS-R. Participants in the two depressed groups had current diagnoses of unipolar depressive disorders on the K-SADS-PL diagnostic interview and had CDRS-R raw scores of 35 or greater. The Depressed without Suicidal Ideation (Dep/SI−) group had scores of zero on the current Severity of Ideation and Intensity of Ideation subscales of the C-SSRS. The Depressed with Suicidal Ideation (Dep/SI+) group consisted of depressed adolescents with current C-SSRS Severity of Ideation and Intensity of Ideation subscale scores of 1 or greater. Exclusion criteria for all participants consisted of lifetime history of mania or psychosis; presence of an active substance use disorder (except nicotine); and any contraindication to magnetic resonance imaging, such as implanted ferromagnetic material or orthodontic hardware that would cause artifact in MRI images.

### ^1^H-MRS methods

All participants underwent structural magnetic resonance imaging and ^1^H-MRS on a General Electric 3 T Discovery 750 scanner (GE Healthcare, Chicago, IL, USA) with an 8-channel head coil and running version 22.1 software. Structural images underwent review for incidental findings by a board-certified neuroradiologist (J.D.P.).

**Fig. 1:**
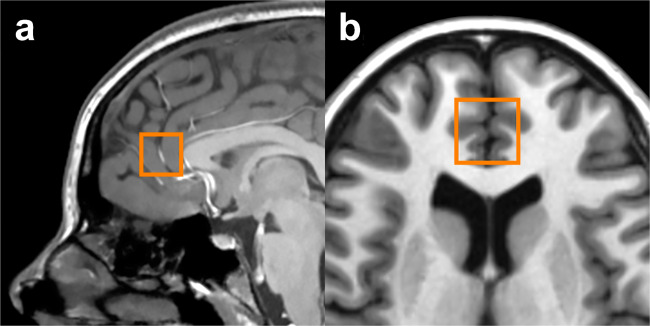
Pregenual anterior cingulate cortex (ACC) voxel. **a** Sagittal view; **b** Axial view.

For acquisition of volumetric data, a FAST 3D SPGR sequence was used (sagittal acquisition, TR = 7.4 ms, TE = 3.0 ms, flip angle = 8°, voxel dimensions = 1.02 × 1.02 × 1.2 mm). Positioning of the ACC voxel was performed according to previously published methods^49^. In brief, an axial reference slice approximately 1 cm superior to the genu of the corpus callosum and permitting a continuous visualization of both anterior and posterior horns of the lateral ventricles was selected. On the reference slice, an 8-cm^3^ (2 × 2 × 2 cm) voxel encompassing predominantly prefrontal gray matter was centered on the interhemispheric fissure, with the posterior margin of the voxel abutting the genu of the corpus callosum. The voxel thus corresponded to the pregenual ACC (Brodmann areas 24a, 24b, and 32 as cytoarchitecturally defined)^50^. Voxel placement is shown in Fig. 1.

Spectroscopic data were acquired using a 2-dimensional *J*-averaged PRESS sequence (TR = 2000 ms, TE = 35–195 ms in 16 steps, TR = 2000 ms, 8 averages, 3-way phase cycling) designed for optimal measurement of glutamate^51,52^. Following the scan, images and spectroscopic data were transferred to a workstation running SAGE-IDL software (GE Healthcare). Integrity of spectra was verified on visual review by the neuroradiologist, and scans with significant visible artifact were excluded. Metabolite concentrations were quantified using LCModel^53^ software version 6.3–1K and a vendor-provided basis set. Scans with signal-to-noise ratios less than 10 were excluded, and individual metabolite measurements were excluded if they had Cramér-Rao lower bounds (representing measurement error) > 20%.

Metabolite concentration measurements were corrected to the cerebrospinal fluid (CSF) fraction according to previously published methods^49,54^. In brief, segmentation of the T2-weighted anatomical images into gray matter, white matter, and CSF was performed using an in-house thresholding technique. The ACC voxel was then superimposed on the segmented anatomical images, and the number of pixels for gray matter, white matter, and CSF were quantified and normalized to the total pixels within the voxel to yield a fraction for each tissue type. The metabolite measurement (M) corrected to the CSF fraction (F_CSF_) was calculated as

$${\mathrm{[M]_{corrected}}} = {\mathrm{[M]_{measured}}} \times \frac{1}{{1 - {\mathrm{F}_{\mathrm{CSF}}}}}$$

and is expressed in institutional units. CSF-corrected Glu, Glx, and NAA concentrations were measured. Additionally, the NAA/Glx ratio was calculated. This was based on prior literature suggesting that the ratio of NAA to glutamatergic concentrations (or vice versa) allows measurement of altered glutamatergic metabolism while correcting for the effect of neuronal loss (as indexed by NAA alone)^55,56^ and may correspond to impairment in the metabolic cycling of glutamate–glutamine–NAA in neuropsychiatric disease states^57,58^.

### Statistical analyses

Statistical analyses were performed using IBM SPSS Statistics for Windows, Version 25 (IBM Corp., Armonk, NY, USA) and JMP Pro 14.1.0 (SAS Institute, Inc., Cary, NC, USA) software. The significance level was set at *α* = 0.05, and *p*-values were adjusted for multiple comparisons according to the false-discovery rate (FDR) method^59^. The normality of distributions for spectroscopic outcome measures (Glu, Glx, NAA, NAA/Glx) was examined with Shapiro-Wilk tests. Distributions of all spectroscopic measures did not deviate from the normal distribution in the overall sample or within any group (all *p* > 0.2). Consequently, parametric statistical tests were used for the analyses.

For our primary aim, a separate fixed-effects general linear model (GLM) was conducted for each spectroscopic measure (Glu, Glx, NAA, NAA/Glx). In each GLM, main effects of the following independent variables were tested: group (HC vs. Dep/SI− vs. Dep/SI+), sex, age at time of scan, and psychotropic medication status (coded as a dichotomous variable denoting the presence or absence of psychotropic medication at the time of the ^1^H-MRS scan). The main effect of group was corrected for a total of four comparisons using the FDR method^59^. Post hoc comparisons of estimated group marginal means on each metabolite (for a total of three pairwise contrasts each) were adjusted for multiple comparisons using the Šidák correction.

For the secondary aim, the relationship between metabolite concentrations and (1) ordinal variables of SI severity, and (2) continuous variables of SI intensity were examined with multiple linear robust regressions that included age, sex, depression severity (adjusted CDRS-R total score), and psychotropic medication status as covariates.

As an exploratory sensitivity analysis, a receiver operating characteristic (ROC) analysis was used to determine the ability of ACC Glx and the NAA/Glx ratio to discriminate between Dep/SI− and Dep/SI+ groups. The analysis tested the areas under the curve (AUCs) of Glx and the NAA/Glx ratio against a nominal AUC of 0.5. The AUCs, 95% confidence intervals, sensitivity, specificity, positive predictive value (PPV), and negative predictive value (NPV) are reported for each optimal cutpoint.

## Results

### Participant characteristics

Forty adolescents (25 female, 15 male; mean age ± SD, 16.66 ± 1.92 years; range 13.58–20.81 years) underwent clinical evaluations and ^1^H-MRS scans. The three groups (HC, Dep/SI−, Dep/SI+ ) did not differ in age or sex distribution. Demographic and clinical characteristics of the three participant groups are reported in Table 1.

**Table 1:** Demographic and clinical characteristics by group.

Characteristic	HC (*n* = 16)	Dep/SI− (*n* = 13)	Dep/SI+ (*n* = 11)	*p*
Age at time of ^1^H-MRS scan (years), mean ± SD	16.90 ± 1.92	16.70 ± 2.08	16.26 ± 1.87	0.708^a^
Sex				0.783^a^
Female, *n* (%)	11 (68.75%)	8 (61.54%)	6 (54.55%)	
Male, *n* (%)	5 (31.25%)	5 (38.46%)	5 (45.45%)	
Family history				
Any psychiatric illness, *n* (%)	7 (50.00%)^b^	13 (100%)	11 (100%)	<0.001^a^
Mood disorder, *n* (%)	7 (50.00%)^b^	13 (100%)	10 (90.91%)	0.003^a^
Attempted or completed suicide, *n* (%)	0 (0%)^b^	5 (41.67%)^c^	7 (63.64%)	<0.001^a^
Depression severity: CDRS-R				
Total score, mean ± SD	18.50 ± 1.41	48.69 ± 7.93	57.64 ± 9.77	0.021^d^
Adjusted total score (without SI, morbid ideation items), mean ± SD	16.50 ± 1.41	45.23 ± 7.24	49.36 ± 9.00	0.225^d^
Current psychotropic medication, *n* (%)	0 (0%)	4 (30.77%)	5 (45.45%)	0.675^d^
Number of depressive episodes, mean ± SD	n/a	1.54 ± 0.66	1.73 ± 0.79	0.529^d^
Duration of current MDE (months), mean ± SD	n/a	5.71 ± 5.42	14.82 ± 17.70	0.127^d^
Cumulative duration of all lifetime MDEs (months), mean ± SD	n/a	11.48 ± 8.26	19.82 ± 16.35	0.120^d^
Duration of depressive illness (years), mean ± SD	n/a	1.59 ± 1.58	2.73 ± 1.98	0.130^d^
Suicidal ideation/behavior: C-SSRS				
Severity of ideation (current), *n* (%)				<0.001^d^
0 = no suicidal ideation	16 (100%)	13 (100%)	0 (0%)	
1 = wish to be dead	0 (0%)	0 (0%)	2 (18.18%)	
2 = non-specific active suicidal thoughts	0 (0%)	0 (0%)	2 (18.18%)	
3 = active suicidal ideation with any method (not plan) without intent to act	0 (0%)	0 (0%)	2 (18.18%)	
4 = active suicidal ideation with some intent to act, without specific plan	0 (0%)	0 (0%)	3 (27.27%)	
5 = active suicidal ideation with specific plan and intent	0 (0%)	0 (0%)	2 (18.18%)	
Intensity of ideation (current), mean ± SD	0 ± 0	0 ± 0	13.09 ± 3.39	<0.001^d^
Maximal lifetime SB severity, *n* (%)				<0.001^d^
0 = none	16 (100%)	11 (84.62%)	0 (0%)	
1 = nonsuicidal self-injurious behavior	0 (0%)	2 (15.38%)	2 (18.18%)	
2 = planning or preparation for attempt	0 (0%)	0 (0%)	0 (0%)	
3 = aborted or interrupted attempt	0 (0%)	0 (0%)	4 (36.36%)	
4 = suicide attempt	0 (0%)	0 (0%)	5 (45.45%)	

^*1*^*H-MRS* proton magnetic resonance spectroscopy, *C-SSRS* Columbia Suicide Severity Rating Scale, *CDRS-R* Children’s Depression Rating Scale, Revised, *Dep/SI−* depressed without suicidal ideation, *Dep/SI+* depressed with suicidal ideation, *HC* healthy control, *MDE* major depressive episode, *n/a* not applicable, *SB* suicidal behavior, *SD* standard deviation, *SI* suicidal ideation.

^a^Three-group comparisons (HC vs. Dep/SI− vs. Dep/SI+) on demographic characteristics were conducted using one-way ANOVAs for continuous variables and Fisher’s exact tests for categorical variables.

^b^Missing two observations.

^c^Missing one observation.

^d^Two-group comparisons (Dep/SI− vs. Dep/SI+) on clinical characteristics were conducted using independent samples *t*-tests for continuous variables and Fisher’s exact tests for categorical variables.

Comparing the two depressed groups, Dep/SI− and Dep/SI+ adolescents did not differ in the proportions of participants who had family histories of psychiatric illness, mood disorders, or attempted or completed suicide (Fisher’s exact tests, *p* > 0.99, *p* = 0.458, *p* = 0.414, respectively). Participants in the Dep/SI+ had higher CDRS-R total scores than did those in the Dep/SI− group (*t* = −2.477, *p* = 0.021). However, the adjusted depression severity score (removing CDRS-R items assessing morbid and suicidal ideation) did not differ (*t* = −1.247, *p* = 0.225). Dep/SI− and Dep/SI+ groups did not differ in number of depressive episodes (*t* = −0.640, *p* = 0.529), current episode duration (*t* = −1.643, *p* = 0.127), cumulative time depressed (*t* = −1.616, *p* = 0.120), or total depressive illness duration (*t* = −1.572, *p* = 0.130). The proportion of participants taking a psychotropic medication did not differ between the two depressed groups (*p* = 0.675). Medications taken by individual participants are reported in Supplemental Table S1.

Among the Dep/SI+ participants, two (18.18%) had current C-SSRS Severity of Ideation scores of 1, two (18.18%) had scores of 2, two (18.18%) had scores of 3, three (27.27%) had scores of 4, and two (18.18%) had scores of 5. On the Intensity of Ideation subscale, scores in the Dep/SI+ group ranged from 8 to 19 (mean score± SD, 13.09 ± 3.39). No adolescents in the HC group had any lifetime suicidal behavior (SB). Two participants in the Dep/SI− group (15.38%) had prior nonsuicidal self-injury; no Dep/SI− adolescents had any history of other forms of SB. In the Dep/SI+ group, two (18.18%) had prior nonsuicidal self-injury, four (36.36%) had a prior aborted or interrupted attempt, and five (45.45%) had made a suicide attempt.

### Primary aim: group comparisons on ^1^H-MRS-measured ACC metabolites

**Fig. 2:**
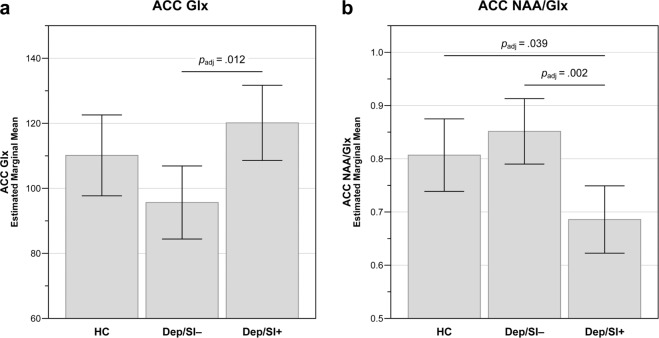
Anterior cingulate cortex (ACC) metabolite differences by group. **a** Glutamate+glutamine (Glx); **b** *N*-acetylaspartate/glutamate+glutamine (NAA/Glx) ratio. General linear models tested main effects of group, covarying for age, sex, and psychotropic medication use. Estimated marginal means and 95% confidence intervals for metabolites are displayed for each adolescent participant group (HC, healthy control, *n* = 16; Dep/SI−, depressed without suicidal ideation, *n* = 13; Dep/SI+ , depressed with suicidal ideation, *n* = 11). *p*-values adjusted for multiple comparisons (*p*_adj_) are displayed for significant group differences in pairwise post hoc tests.

Estimated marginal means of ACC metabolites are reported in Table 2. In the GLM analyses, there were no significant main effects of group (HC vs. Dep/SI− vs. Dep/SI+ ) on Glu (*F*_2,32_ = 1.888, *p* = 0.168, *p*_FDR _= 0.178, $\eta _{p}^2$ = 0.106) or NAA (*F*_2,32_ = 1.821, *p* = 0.178, *p*_FDR_ = 0.178, $\eta _{p}^2$ = 0.102). However, there were significant group main effects for ACC Glx (*F*_2,31_ = 5.003, *p* = 0.013, *p*_FDR _= 0.026, $\eta _{p}^2$ = 0.244) and the ACC NAA/Glx ratio (*F*_2,31_ = 7.473, *p* = 0.002, *p*_FDR_ = 0.008, $\eta _{p}^2$ = 0.325). In post hoc pairwise contrasts (Fig. 2), Dep/SI− participants had lower mean ACC Glx than Dep/SI+ adolescents (*p*_adj_ = 0.012), although they did not differ from HC participants (*p*_adj _= 0.190), and HC and Dep/SI+ adolescents also did not differ in mean ACC Glx (*p*_adj _= 0.567). Dep/SI+ adolescents had a significantly lower mean NAA/Glx ratio than both Dep/SI− participants (*p*_adj_ = 0.002) and HC adolescents (*p*_adj _= 0.039), whereas Dep/SI− and HC participants did not differ (*p*_adj_ = 0.649).

**Table 2:** ^1^H-MRS-measured anterior cingulate metabolites by group.

Metabolite	Estimated marginal mean ±SE			*p* (*p*_FDR_)	$\eta _{p}^2$
	HC (*n* = 16)	Dep/SI− (*n* = 13)	Dep/SI+ (*n* = 11)		
Glu	85.04 ± 4.19	78.44 ± 3.71^a^	88.43 ± 3.93^a^	0.168 (0.178)	0.106
Glx	110.13 ± 6.10	95.64 ± 5.51^b^	120.13 ± 5.67^a^	0.013 (0.026)	0.244
NAA	85.74 ± 2.40	80.39 ± 2.12^a^	81.07 ± 2.25^a^	0.178 (0.178)	0.102
NAA/Glx	0.807 ± 0.033	0.852 ± 0.030^b^	0.686 ± 0.031^a^	0.002 (0.008)	0.325

General linear models (GLMs) were used to examine the main effect of group on metabolite concentrations, covarying for sex, age at scan, and psychotropic medication use. *p*-values and reported effect sizes ($\eta _{p}^2$) are for main effect of group.

^*1*^*H-MRS* proton magnetic resonance spectroscopy, *Dep/SI−* depressed without current suicidal ideation, *Dep/SI+* depressed with current suicidal ideation, *Glu* glutamate, *Gl*x glutamate+glutamine, *HC* healthy control, *NAA* *N*-acetylaspartate, *p*_FDR_ *p*-value adjusted for multiple comparisons according to the false-discovery rate procedure^59^, *SE* standard error of the mean.

^a^Missing one observation (Cramér-Rao lower bound > 20%).

^b^Missing two observations (Cramér-Rao lower bound > 20%).

### Secondary aim: relationships between glutamatergic neurochemistry and suicidal ideation

In the multiple linear regression analyses (Table 3), adjusting for age, sex, psychotropic medication, and depression severity (adjusted CDRS-R total score), there were no significant relationships between severity of SI (C-SSRS Severity of Ideation subscale) and ACC Glu (*b̂* = 2.486, *p* = 0.140, *p*_FDR _= 0.187), Glx (*b̂* = 5.653, *p* = 0.032, *p*_FDR_ = 0.064), or NAA (*b̂* = −0.262, *p* = 0.781, *p*_FDR _= 0.781). However, there was a significant negative relationship between SI severity and ACC NAA/Glx (*b̂* = −0.044, *p* = 0.003, *p*_FDR _= 0.012).

**Table 3:** Relationships between suicidal ideation and ^1^H-MRS-measured anterior cingulate metabolites.

Metabolite	*b̂*	SE	95% CI for *b̂*	*p* (*p*_FDR_)
	Severity of ideation			
Glu	2.486	1.644	−0.863 to 5.834	0.140 (0.187)
Glx	5.653	2.518	0.518 to 10.788	0.032 (0.064)
NAA	−0.262	0.936	−2.168 to 1.644	0.781 (0.781)
NAA/Glx	−0.044	0.014	−0.072 to −0.016	0.003 (0.012)
	Intensity of ideation			
Glu	0.655	0.403	−0.167 to 1.476	0.114 (0.152)
Glx	1.603	0.607	0.365 to 2.841	0.013 (0.026)
NAA	−0.014	0.231	−0.484 to 0.457	0.952 (0.952)
NAA/Glx	−0.012	0.003	−0.018 to −0.005	0.001 (0.004)

Multiple linear regressions examining relationships of suicidal ideation severity and intensity with ^1^H-MRS-measured ACC metabolites. Regression models include sex, age at scan, psychotropic medication use, and depression severity (adjusted CDRS-R total score). The unstandardized parameter estimate (coefficient) for the SI variable (*b̂*), standard error of *b̂*, 95% confidence intervals for *b̂*, and *p*-values for the regression relationship between the suicidal ideation variable and the ACC metabolite are reported.

^*1*^*H-MRS* proton magnetic resonance spectroscopy, *CI* confidence interval, *Glu* glutamate, *Glx* glutamate+glutamine, *NAA* *N*-acetylaspartate, *p*_FDR_ *p*-value adjusted for multiple comparisons according to the false-discovery rate procedure^59^, *SE* standard error.

In separate multiple linear regression models, again adjusting for age, sex, psychotropic medication, and depression severity (adjusted CDRS-R total score), there were no significant relationships between SI intensity (C-SSRS Intensity of Ideation subscale) and ACC Glu (*b̂* = 0.655, *p* = 0.114, *p*_FDR _= 0.152) or NAA (*b̂* = −0.014, *p* = 0.952, *p*_FDR _= 0.952). There was a significant positive relationship between SI intensity and Glx (*b̂* = 1.603, *p* = 0.013, *p*_FDR_ = 0.026), as well as a significant negative relationship between SI intensity and ACC NAA/Glx (*b̂* = −0.012, *p* = 0.001, *p*_FDR _= 0.004).

### Exploratory sensitivity analyses

The ROC analysis indicated that ACC Glx, using a cutpoint of ≥ 109.811, discriminated Dep/SI− participants from Dep/SI+ participants (AUC = 0.864, SE = 0.083, *p* = 0.005, *p*_FDR _= 0.005) with 90.00% sensitivity, 81.80% specificity, a PPV of 81.82%, and an NPV of 90.00%. The ROC analysis determined that the ACC NAA/Glx ratio, using a cutpoint of ≤ 0.73995, discriminated Dep/SI− participants from Dep/SI+ participants (AUC = 0.900, SE = 0.066, *p* = 0.002, *p*_FDR _= 0.004) with 81.80% sensitivity, 80.00% specificity, a PPV of 80.00%, and an NPV of 81.82%. ROC curves for the ACC Glx and NAA/Glx sensitivity analyses are displayed in Supplemental Fig. S1.

## Discussion

The ACC plays essential roles in cognitive and emotional processes relevant to suicidal ideation and behavior. Through its prefrontal and limbic projections, the ACC mediates input from executive functions and motivational drives^39,60^. The dorsal/caudal ACC has been implicated in attentional and interpretive mechanisms used in evaluating internal and external stimuli^61,62^. The rostral (pregenual and subgenual) ACC, a portion of which was sampled in our study, is involved in affective regulation via inhibition of limbic and sympathetic responses to negatively valent stimuli and emotional conflict^43,62^. This latter ACC division, in conjunction with other areas of the medial prefrontal cortex, also appears to be involved in determining self-relevance of emotionally salient stimuli^40,41^. Encoding emotional valence involves implicit cognitive associations^63^, which have been shown to involve ACC activity in electroencephalographic^63^ and functional MRI^64^ studies. Implicit associations to suicide- and self-injury-related stimuli are stronger in adults^65^ and adolescents^66–68^ with histories of suicidality, and experimentally measured suicide- and self-injury-related implicit associations predict future suicidal ideation and self-harm in adolescents^66,67,69^. Altered rostral ACC activity also has been linked to other characteristics of suicidal individuals, including rumination and negative self-referential thinking^42–44^. Moreover, task-related ACC activation^70–72^ and functional connectivity to other emotion-regulating regions^71^ differ between adolescent suicide attempters and depressed non-attempters, suggesting that suicidality may involve ACC functions distinct from those related to depressive mood states.

Spectroscopic studies comparing depressed and healthy adolescents have found glutamatergic deficits in the ACC^33–35^ and diminished NAA concentrations in ACC and medial prefrontal cortex^36^, although, to our knowledge, none have directly compared cortical neurochemical profiles of suicidal and nonsuicidal youth. In adults, prior ^1^H-MRS studies have shown mixed findings on the potential roles of cortical glutamate and NAA in suicidality. Sheth et al.^73^ found no differences in Glu/H_2_O or NAA/H_2_O concentrations in dorsal ACC and posterior cingulate voxels between groups of military veterans with and without suicidal behavior (SB). In the same sample, Prescot et al.^74^ examined dorsal ACC metabolite concentrations in the overall sample, as well as in male and female subgroups; no differences in NAA/Cr+PCr or Glu/Cr+PCr were observed between veterans with and without SB in the overall sample, or within either sex group. Other ^1^H-MRS studies have examined potential relationships between suicidality and cortical metabolism in regions beyond the ACC. Jollant et al.^32^ utilized ^1^H-MRS to examine metabolites in the right dorsolateral prefrontal cortex (DLPFC) in healthy and depressed adults, including those with historical SB. Although no group differences survived correction for multiple comparisons, healthy control adults had lower Gln than both nonsuicidal depressed and suicide attempter groups, while NAA was lower in suicide attempters than in healthy controls. Right DLPFC NAA concentrations correlated negatively with current psychological pain, which persisted when controlling for various clinical characteristics such as age, gender, and depression severity; additionally, psychological pain mediated the relationship between DLPFC NAA and current SI^32^. Smesny et al.^75^ examined metabolite concentrations in adults with cluster B and C personality disorders, conditions with increased suicide risk, and in healthy comparators. The investigators found increased right DLPFC Glu and decreased right dorsal ACC NAA in cluster B patients, while cluster C patients demonstrated decreased NAA in bilateral DLPFC, left dorsomedial prefrontal cortex, and left dorsal ACC voxels, as well as decreased bilateral DLPFC and left dorsomedial prefrontal Glu^75^. By contrast, Rocha et al.^31^ found no difference in orbitofrontal cortical metabolite concentrations between healthy adults and groups of currently euthymic bipolar patients with and without historical suicide attempts.

When considering our results in the context of these earlier, disparate findings, it is important to note certain methodological differences. First, less mature excitatory circuitry in our younger sample may contribute to the differences in metabolite measurements between groups that we observed compared with prior adult studies. Second, metabolite concentrations differ not only between brain regions but also between heterogeneous segments of a single structure; indeed, many studies that sampled ACC voxels^73–75^ examined more dorsal aspects of the ACC than our pregenual voxel. The pregenual ACC is distinct in glutamatergic receptor density and microarchitecture compared to other ACC subregions, and prior ^1^H-MRS work has found the pregenual ACC to have higher Glu and Gln concentrations than more caudal subregions^76^. Furthermore, many prior studies have referenced metabolite values to total creatine (Cr+PCr). Total creatine has been found to differ in a variety of neuropsychiatric disease states in both adults^54,77^ and youth^78,79^. Thus, studies reporting metabolite concentrations relative to Cr+PCr introduce this additional confound when comparing clinical and healthy groups, and creatine-referenced metabolite values may not be directly comparable to CSF-corrected absolute concentrations as measured in our study.

Additionally, many prior studies classified patients on the basis of having a history of SB. Despite the frequent presence of prior SB in persons with current SI, it has not been established whether the neurochemical correlates of historical behavior are necessarily the same as those of current ideation. The grouping of depressed participants by presence or absence of current SI in our study is unique, and future work with larger samples of current ideators with and without prior SB is necessary to determine whether their neural metabolite profiles differ.

The most novel findings in our study were that the ACC NAA/Glx ratio was reduced in Dep/SI+ adolescents compared to those in the HC and Dep/SI− groups, correlated with current SI intensity and severity, and significantly discriminated depressed adolescents with and without current SI. Notably, these findings were observed in the absence of significant group differences in NAA or significant relationships between SI and NAA. This raises important questions about the meaning of the NAA/Glx ratio and the role that dysregulated NAA−glutamate metabolism in this crucial brain region might play in suicidality. NAA is found predominantly in neurons, and ^1^H-MRS-measured NAA values correspond to neuronal density^80,81^. Diminished NAA has been found in disease processes involving neuronal loss, and yet NAA concentrations also have been observed to recover, suggesting that NAA may index both permanent and state-dependent aspects of neuronal health, viability, and activity^80–82^. The ^1^H-MRS-measured Glx concentration is a composite of Glu and Gln signals, with γ-aminobutyric acid (GABA) and glutathione also being minor factors^80,81^. Glutamate both functions as the main excitatory transmitter and has roles in energy metabolism, while glutamine serves predominantly as an intermediate for glutamate and GABA synthesis, being shuttled between astrocytes and neurons in a form less reactive than these transmitters^81^. Glx thus indicates the combined (neuronal and glial) cytosolic pool of glutamate and glutamine that can be used for both energetic and neurotransmission functions^80,81^. Neuronal NAA can be converted to glutamate via a series of reactions occurring in astrocytes, oligodendrocytes, and neuronal mitochondria, and thus NAA also may serve as a reservoir for the production of glutamate and glutamine, particularly in conditions of metabolic stress^57^. NAA may index ATP-dependent metabolism, as well as this alternative glutamate-dependent energy production, in neuronal mitochondria^82^. Considering the complex relationships between these metabolites, examining their concentrations relative to one another, as indicated by a ratio, may offer insights into how cycling and metabolism of glutamate, glutamine, and NAA differ in pathological conditions^57,58^. Additionally, concentration ratios may be more sensitive to metabolic derangements of related molecules than single metabolite measurements alone^55,83^.

Local dysregulation in NAA and glutamatergic metabolism, as indicated by low NAA/Glu or NAA/Glx concentrations (or, inversely, high Glu/NAA or high Glx/NAA), has been identified as a potential marker of damage to brain structures or networks that correspond to the symptomatic processes specific to diverse neurologic and psychiatric conditions. White matter Glu/NAA was elevated in a large sample of patients with multiple sclerosis compared to healthy controls, and this ratio predicted longitudinal brain volume loss^55^. Hypothalamic Glx/NAA also was higher in multiple sclerosis patients than in healthy comparators, and was higher in patients with more active disease, while Glx/NAA also corresponded to symptom severity and fatigue^58^. Primary motor cortex NAA/Glu correlated negatively with disease duration in amyotrophic lateral sclerosis^84^. Glx/NAA was elevated in epileptogenic foci relative to healthy brain regions in partial epilepsy, and demonstrated utility in identifying seizure foci^56^. In schizophrenia, hippocampal Glx/NAA was increased compared to healthy adults^83^. Significant positive correlations between Glx and NAA concentrations were present in healthy controls but not in schizophrenic patients in the hippocampus^83,85^ right DLPFC^86^, and left striatum^87^, suggesting that the usually linked metabolism of NAA and glutamate becomes uncoupled in these regions in the disease state. Similarly, right hippocampal Glu/NAA was elevated in posttraumatic stress disorder (PTSD) patients compared to trauma-exposed controls, which correlated with re-experiencing symptoms and trauma load in patients^88^. It is notable that the symptomatology of both schizophrenia and PTSD are characterized by deficits in processes involving the hippocampus, and that the DLPFC and striatum have been implicated previously in schizophrenia. Altered NAA−glutamate metabolism in the ACC, by comparison, might be expected to correspond to conditions typified by impaired emotion-processing functions, such as mood disorders and suicidality. In one study, ACC NAA/Glx was lower in adults with bipolar disorder compared to healthy controls, both before treatment and after 12 weeks of lamotrigine^89^. This suggests the need to examine ACC glutamatergic metabolism in suicidal individuals in longitudinal studies, both for changes that occur in conjunction with natural fluctuations in suicidal risk and also for the effects (or lack thereof) of anti-suicidal interventions.

Our study has several important limitations. The sample was small, and larger, well-powered investigations are necessary to replicate these findings before they can inform clinical risk assessment and future interventions. It is particularly important for future studies to include adequate numbers of both male and female participants across broad neurodevelopmental trajectories. NAA and glutamate levels have been found to differ between age and sex groups among healthy individuals^90^, and patterns of suicidal behavior differ by both age and sex. The limited research examining sex-related differences in excitatory−inhibitory neurochemistry in suicidality suggests potential distinctions in ACC metabolism, but these remain poorly understood^74^. Regarding our spectroscopic methodology, the 2-dimensional *J*-averaged PRESS sequence used in this study is optimized for glutamate signal acquisition at 3 T, but it did not permit reliable measurement of Gln or GABA. It is noteworthy that while we did find significant group differences and a relationship with SI for ACC Glx, there were no significant findings for Glu. This suggests the possibility that glutamine, which accounts for the majority of the non-Glu portion of the Glx signal, could be responsible for the discrepancy between our Glu and Glx findings. This is particularly relevant considering that the pregenual ACC has a substantially higher ratio of Gln to Glu than other subregions of the cingulate gyrus^76^. Moreover, preliminary data suggest that these related metabolites are productive areas for further study of the intersection of mood disorders and suicidality. For example, DLPFC Gln was lower in healthy adults compared to depressed adults with and without prior suicide attempts^32^, and ACC GABA concentrations were found to be reduced in female veterans with SB and correlated negatively with measures of suicidality^74^. Further ^1^H-MRS studies are needed to understand how the metabolically-linked GABA and glutamate−glutamine systems relate to clinical features such as SI and SB. TE-optimized PRESS approaches^91^ that allow accurate measurement of Gln and MEGA-PRESS sequences designed to quantify GABA^92,93^, in conjunction with the use of higher field strengths, may yield more comprehensive insights into how cortical excitatory−inhibitory metabolism corresponds to acute suicidality. Furthermore, the instrument used for classifying participants and rating the intensity and severity of SI in our study, the C-SSRS, was designed to assess clinical suicide risk. Future investigations should utilize dimensional measures assessing not only overt measures of suicidality, but also specific cognitive and emotional constructs associated with suicidal thoughts and behaviors. Several promising adolescent studies have examined the role of cortical neurochemistry in symptomatologic features relevant to suicidality. Anhedonia has demonstrated strong associations with suicidality that are independent of other depressive symptoms^94–97^. Pregenual ACC Gln was lower in highly anhedonic depressed adults than in low-anhedonia depressed and healthy individuals, whereas ACC glutamate and NAA correlated with functional MRI-measured ACC activation in response to emotional stimuli in depressed persons^98^. By contrast, ACC GABA was found to be significantly reduced in anhedonic depressed youth compared to nonanhedonic depressed and healthy adolescents^37,38^. However, the relationships between constructs like anhedonia and complex behaviors like SB remain poorly understood at present. Future research must strive to delineate how symptom-related, cognitive, and emotional functions mediate the relationship between SB and observed neurochemical deficits in particular brain structures and circuits. Doing so will enable the development of more comprehensive and sophisticated models of the neurobiology of suicidality.

## Supplementary information

Figure S1

Table S1.
