# Supplementary figures and images for: Altered anterior cingulate glutamatergic metabolism in depressed adolescents with current suicidal ideation
Source: Transl Psychiatry. 2020 Apr 23;10:119. doi: 10.1038/s41398-020-0792-z (PMC7181616; doi:10.1038/s41398-020-0792-z)

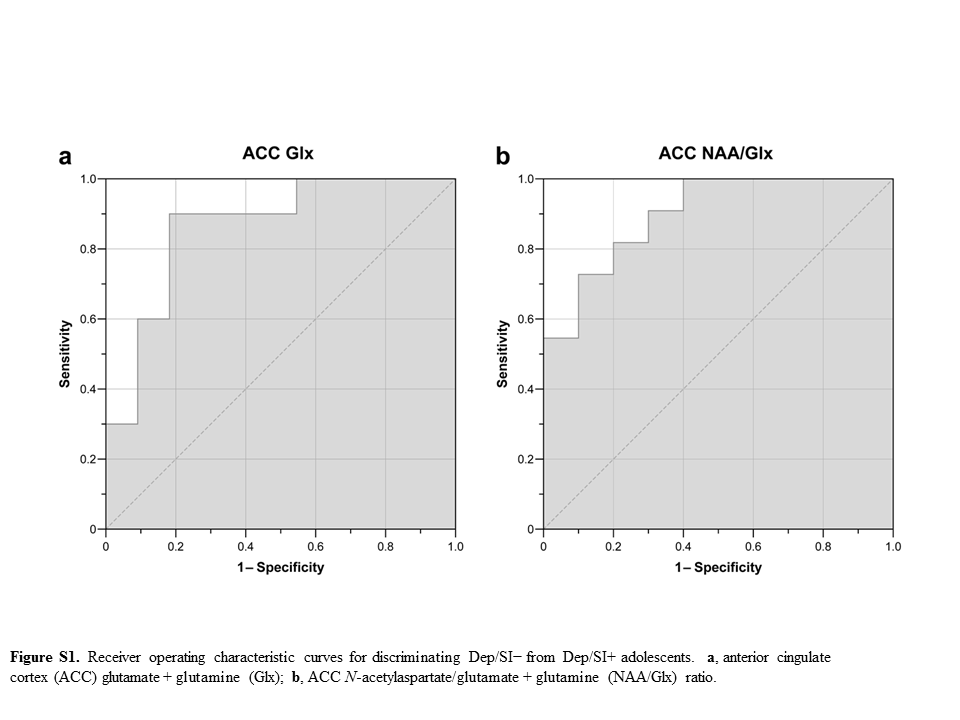

Supplement: Supplementary file 1 — Figure S1 [file 41398_2020_792_MOESM1_ESM.tif]
