# Supplementary material for: Altered anterior cingulate glutamatergic metabolism in depressed adolescents with current suicidal ideation
Source: Transl Psychiatry. 2020 Apr 23;10:119. doi: 10.1038/s41398-020-0792-z (PMC7181616; doi:10.1038/s41398-020-0792-z)
Supplement: Supplementary file 2 — Table S1. [file 41398_2020_792_MOESM2_ESM.docx]

| **Participant** | **Group** | **Medication(s) and Total Daily Dose** |
| --- | --- | --- |
| 1 | Dep/SI− | none |
| 2 | Dep/SI− | fluoxetine 30 mg |
| 3 | Dep/SI− | none |
| 4 | Dep/SI− | none |
| 5 | Dep/SI+ | escitalopram 10 mg |
| 6 | Dep/SI+ | sertraline 100 mg |
| 7 | Dep/SI+ | none |
| 8 | HC | none |
| 9 | HC | none |
| 10 | HC | none |
| 11 | HC | none |
| 12 | Dep/SI+ | none |
| 13 | HC | none |
| 14 | Dep/SI+ | sertraline 150 mg, aripiprazole 2 mg |
| 15 | Dep/SI+ | fluoxetine 40 mg |
| 16 | HC | none |
| 17 | HC | none |
| 18 | Dep/SI− | none |
| 19 | Dep/SI− | none |
| 20 | HC | none |
| 21 | Dep/SI+ | none |
| 22 | Dep/SI+ | none |
| 23 | Dep/SI− | none |
| 24 | Dep/SI− | none |
| 25 | Dep/SI+ | fluoxetine 20 mg, amphetamine-dextroamphetamine 30 mg^1^ |
| 26 | Dep/SI+ | none |
| 27 | Dep/SI+ | none |
| 28 | HC | none |
| 29 | HC | none |
| 30 | Dep/SI− | duloxetine 120 mg |
| 31 | Dep/SI− | none |
| 32 | Dep/SI− | venlafaxine 150 mg |
| 33 | HC | none |
| 34 | HC | none |
| 35 | Dep/SI− | none |
| 36 | HC | none |
| 37 | Dep/SI− | amitriptyline 50 mg |
| 38 | HC | none |
| 39 | HC | none |
| 40 | HC | none |
| ^1^ Stimulant medications held on day of ^1^H-MRS scan. | | |
